# Acute-Phase Initiation of Cardiac Rehabilitation for Short-Term Improvement in Activities of Daily Living in Patients Hospitalized for Acute Heart Failure

**DOI:** 10.3390/jcdd9040097

**Published:** 2022-03-25

**Authors:** Kensuke Ueno, Kentaro Kamiya, Hidehiro Kaneko, Akira Okada, Hidetaka Itoh, Katsuhito Fujiu, Norifumi Takeda, Hiroyuki Morita, Nobuaki Michihata, Taisuke Jo, Hideo Yasunaga, Issei Komuro

**Affiliations:** 1 Department of Cardiovascular Medicine, The University of Tokyo, Tokyo 113-8655, Japan; ap16309@st.kitasato-u.ac.jp (K.U.); hidehikaneko-circ@umin.ac.jp (H.K.); hitoh.ggl@gmail.com (H.I.); fujiu-tky@g.ecc.u-tokyo.ac.jp (K.F.); norifutakeda@gmail.com (N.T.); hiroymorita@gmail.com (H.M.); komuro_tky2000@yahoo.co.jp (I.K.); 2 Department of Rehabilitation Sciences, Graduate School of Medical Sciences, Kitasato University, Sagamihara 252-0373, Japan; 3 Department of Rehabilitation, School of Allied Health Sciences, Kitasato University, Sagamihara 252-0373, Japan; 4 Department of Advanced Cardiology, The University of Tokyo, Tokyo 113-8655, Japan; 5 Department of Prevention of Diabetes and Lifestyle-Related Diseases, Graduate School of Medicine, The University of Tokyo, Tokyo 113-8655, Japan; aokada@m.u-tokyo.ac.jp; 6 Department of Health Services Research, The University of Tokyo, Tokyo 113-0033, Japan; gha10771@gmail.com (N.M.); jo.taisuke@gmail.com (T.J.); 7 Department of Clinical Epidemiology and Health Economics, School of Public Health, The University of Tokyo, Tokyo 113-0033, Japan; yasunagah@m.u-tokyo.ac.jp

**Keywords:** cardiac rehabilitation, acute heart failure, early rehabilitation, activities of daily living

## Abstract

Background: Whether acute-phase cardiac rehabilitation (CR) is beneficial for short-term improvement in activities of daily living (ADL) in patients hospitalized for acute heart failure (AHF) remains unclear. Aim: To investigate the association of acute-phase initiation of CR with short-term improvement in ADL in patients hospitalized for AHF. Methods: We retrospectively analyze data from the Diagnosis Procedure Combination Database, a nationwide inpatient database. Patients hospitalized for HF between January 2010 and March 2018 are included. Propensity score matching and generalized linear models are built to examine the association between improvement in ADL and acute-phase CR initiation, defined as the initiation of CR within two days of admission. Results: Among 306,826 eligible patients, CR is initiated in 45,428 patients (14.8%) within two days of hospital admission. Propensity score matching creates 45,427 pairs. CR initiation within two days of hospital admission is associated with ADL improvement (risk ratio: 1.018; 95% confidence interval: 1.004–1.032), particularly in elderly patients, females, and individuals with low ADL at admission, body mass index of 18.5–24.9 kg/m^2^, and New York Heart Association class IV. Conclusions: Our analyses highlight the possibility that acute-phase CR initiation may result in short-term improvement in ADL in patients hospitalized for AHF.

## 1. Introduction

Acute heart failure (AHF) is one of the leading causes of unexpected hospitalization [1], and it is associated with a deterioration in health-related quality of life, frequent rehospitalization, and high mortality [2,3,4]. The benefits of cardiac rehabilitation (CR) for patients with AHF are receiving much attention lately, and a recent report showed that acute-phase CR initiation is associated with better short-term clinical outcomes in patients with AHF [5]. AHF is reported to present with a severe physical dysfunction and impairment of activities of daily living (ADL) [6,7,8,9]. Physical dysfunction and ADL impairment are major determinants of adverse outcomes, such as high readmission and mortality rates in patients with AHF [7,10,11]. In some patients with heart failure (HF), ADL impairment may not improve or may become worse during hospitalization [12]. Recent studies of patients requiring intensive care have reported that early rehabilitation intervention is associated with better functional recovery [13,14,15], but whether acute-phase CR initiation contributes to short-term ADL improvement in patients with AHF has not been adequately investigated. In this study, we aimed to investigate the association of acute-phase CR initiation with improvement in ADL in patients hospitalized for acute HF using a nationwide inpatient database. Further, we examined whether patient characteristics influenced the effect of acute-phase CR initiation on ADL improvement. We believe that this study provides clinical value at a time when CR in patients with AHF is in the spotlight and is widely used in clinical practice.

## 2. Materials and Methods

### 2.1. Study Design and Data Source

We performed a retrospective cohort study using data from Diagnosis Procedure Combination database, a nationwide inpatient database in Japan. The data collected included administrative claims and clinical data of approximately 8 million hospitalized patients every year from more than 1200 participating hospitals, including 82 academic hospitals [16,17,18,19,20,21]. These hospitals are distributed across 47 prefectures in Japan. The Diagnosis Procedure Combination database represents approximately 50% of all acute inpatients and covers more than 90% of all tertiary-care emergency hospitals in Japan. Academic hospitals are required to contribute to this database. However, the participation of community hospitals is voluntary. The database collates the main diagnoses, comorbidities present at admission, and complications during hospitalization using the International Classification of Diseases and Related Health Problems 10th Revision codes (ICD-10 codes). In this study, the diagnosis of HF was made using ICD-10 codes. Especially, we identified patients admitted and discharged with a primary discharge diagnosis using the ICD-10 codes I50.0, I50.1, and I50.9 between January 2010 and March 2018. Information on treatments, including medication use, procedures, and CR, is also available.

This study was approved by the Institutional Review Board of the University of Tokyo (Approval number: 3501-[3]) and was conducted in accordance with the tenets of the Declaration of Helsinki. Based on the anonymous nature of the data used, the need for obtaining informed consent was waived.

### 2.2. Participants

We studied 447,818 patients aged ≥ 20 years with a length of stay ≥ 3 days and New York Heart Association (NYHA) class ≥ II who had not undergone major procedures under general anesthesia. Patients meeting the following criteria were excluded: requirement of advanced mechanical support within 2 days after admission, including intubation, intra-aortic balloon pumping, and extracorporeal membrane oxygenation (ECMO) (*n* = 9040); Japan Coma Scale 2 digits (*n* = 6476), 3 digits (*n* = 2077), and missing (*n* = 9); missing body mass index (BMI) (*n* = 37,040), Barthel index (BI) at admission (*n* = 69,104), and BI on hospital discharge (*n* = 17,246) data.

Acute-phase CR was defined as the initiation of CR within 2 days of admission because acute-phase rehabilitation was defined as the initiation within 2 days of admission in many studies and has been shown to be effective [5,13].

### 2.3. Outcomes

The primary outcome was short-term improvement in ADL between admission and hospital discharge for each patient. ADL evaluation was performed by calculating the BI. The BI consists of 10 items (i.e., bowels, bladder, grooming, toilet use, feeding, transfer, mobility, dressing, stairs, and bathing) to evaluate the functional ability to perform basic ADL [22]. Patients who died during hospitalization were assigned a score of 0 for BI on hospital discharge [23]. In this study, to examine the short-term effect of acute-phase rehabilitation on improvement in ADL, the difference in BI between admission and discharge was calculated. A BI difference ≥ 1 point was defined as an improvement in ADL, while a BI difference ≤ 0 point was defined as non-improvement [16].

### 2.4. Statistical Procedures

Categorical and continuous data are presented as percentages (%) and means (standard deviations [SDs]), respectively. Categorical and continuous variables were compared between patients with and without acute-phase CR initiation using chi-square and unpaired *t*-tests, respectively. The *p*-values < 0.05 were considered statistically significant.

### 2.5. Propensity Score Matching

We performed propensity score matching using a 1:1 matching protocol width equal to 0.2 SD of the logit of the propensity scores, to account for differences in baseline clinical characteristics. We estimated the propensity scores by fitting a logistic regression model for receipt of acute-phase CR initiation as a function of patient demographics. The demographic parameters included the following: age, sex, BMI, atrial fibrillation, hypertension, diabetes mellitus, chronic renal failure, chronic liver disease, chronic respiratory disease, anemia, cancer, myocardial infarction, dilated cardiomyopathy, smoking, prior hospital admission, NYHA class, total BI score at admission, BI sub-item score (i.e., bowels, bladder, grooming, toilet use, feeding, transfer, mobility, dressing, stairs, and bathing) at admission, consciousness level (Japan Coma Scale), weekend admission, medications within 2 days after admission including beta blocker, renin-angiotensin system inhibitor, mineralocorticoid receptor antagonist, tolvaptan, intravenous inotropic agent, intravenous nitrate, intravenous carperitide, intravenous furosemide, procedures within 2 days after admission (including respiratory support and hemodialysis), intensive care unit stay within 2 days after admission, educational institute, hospital volume, and year of admission. The association of acute-phase CR initiation with ADL improvement was evaluated using a generalized linear model. To examine which patients benefited the most regarding improvement in ADL, we stratified the patients by their baseline clinical characteristics and performed propensity score matching in each, which was evaluated using a generalized linear model. The baseline clinical characteristics were as follows: BI at admission, age, sex, BMI, and NYHA class.

### 2.6. Sensitivity Analysis

We conducted three sensitivity analyses to confirm the robustness of the results of this study. First, we re-defined acute-phase CR initiation as the initiation of CR within 3 days after hospital admission according to previous studies [15,16]. Patients with a length of stay of 3 days were excluded from the analysis to consider immortal time bias. Then, we conducted another propensity score matching analysis under this definition. Second, we performed the inverse probability of treatment weighting (IPTW) to account for differences in baseline clinical characteristics using the definition of acute-phase initiation of CR within 2 days of admission. The propensity score used in IPTW was the same as that used in propensity score matching. We used the stabilized average treatment effect weight. This allowed us to maintain the total sample size of the original data and allowed for a more accurate interval estimate of variance of the main effect and control for type I error than the non-stabilized IPTW [24]. A weighted generalized linear model was used to examine the association between acute-phase CR initiation with ADL improvement. Third, IPTW and weighted generalized linear models were performed with the definition of acute-phase initiation of CR as within 3 days of admission.

All statistical analyses were performed using SPSS software version 25 (IBM, Armonk, NY, USA) and Stata version 17 (StataCorp, College Station, TX, USA).

## 3. Results

### 3.1. Study Population

**Figure 1:**
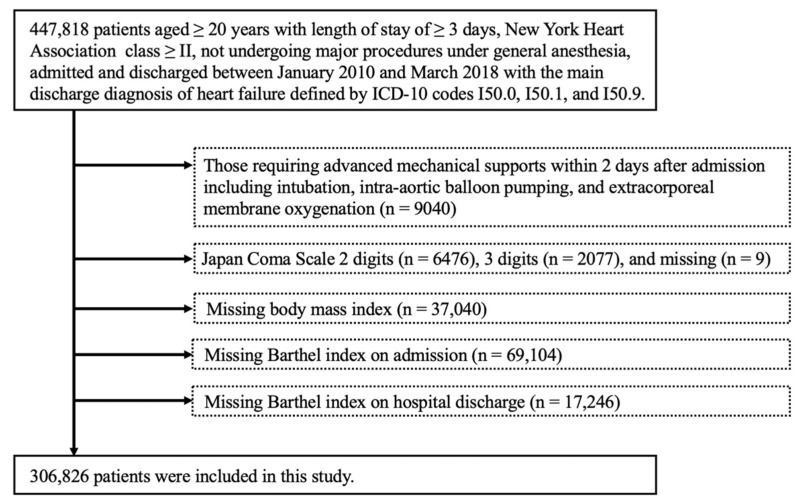
Flow chart of patient selection.

Of the 447,818 patients studied in this study, 140,992 patients were excluded based on the exclusion criteria, resulting in an analysis using data from 306,826 patients (Figure 1). Among these, CR was performed in 142,590 patients (46.5%), 45,428 (14.8%) of whom received acute-phase CR initiation (CR initiation within 2 days after admission). The median time from admission to CR initiation intervention was 4 days (2–8 days).

### 3.2. Characteristics of Study Population

Table 1 shows the characteristics of the study population. Patients with acute-phase CR initiation were more likely to be older, female, and have a lower BI at admission compared to those without. After 1:1 propensity score matching, 45,427 pairs were matched, and both groups were well balanced.

### 3.3. Propensity Score Analyses

**Figure 2:**
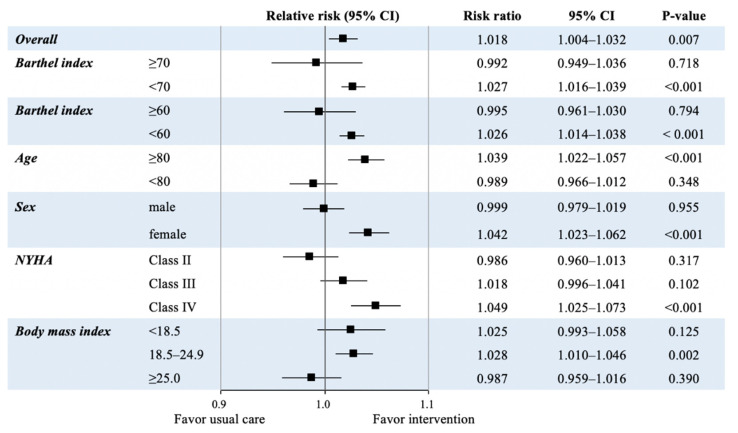
Risk ratios of acute-phase initiation of cardiac rehabilitation to improve activities of daily living in the propensity score matched cohort. BMI: body mass index; CI: confidence interval; NYHA: New York Heart Association.

Figure 2 shows the primary outcomes of the study population, summarizing the risk ratio (RR) of acute-phase CR initiation for improvement of ADL. Overall, acute-phase initiation of CR was associated with ADL improvement (RR, 1.018; 95% confidence interval [CI], 1.004–1.032; *p* = 0.007). Moreover, acute-phase CR initiation was associated with improvement of ADL in patients with a low ADL (BI < 70 and BI < 60) at admission, older age (≥80), female, standard body weight (BMI 18.9–24.9), and NYHA class IV.

### 3.4. Sensitivity Analysis

In the sensitivity analysis, 60,358 well-balanced pairs were matched after 1:1 propensity score matching (Supplemental Table S2). CR initiation within 3 days after hospital admission was associated with improvement of ADL (RR, 1.028; 95% CI, 1.016–1.040; *p* < 0.001) (Table 2). In addition, after the IPTW, both groups were well-balanced with both definitions of acute-phase CR initiation within 2 and 3 days after hospital admission (Supplemental Tables S3 and S4). After IPTW, acute-phase CR initiation was associated with improvement in ADL (Table 2).

## 4. Discussion

The present study used a nationwide inpatient database to examine whether acute-phase CR initiation was associated with short-term improvement in ADL in patients hospitalized for AHF. Our results revealed that acute-phase CR initiation was associated with improvement in ADL after propensity score matching, but the effect size (RR, 1.018; 95% CI, 1.004–1.032) was smaller than anticipated. However, this positive association was consistent even in models using IPTW and when we defined acute-phase CR initiation as within 3 days after admission. Additionally, we found that in a sub-population, such as in elderly patients, females, patients with low ADL (BI < 70 and <60) at admission, those with BMI of 18.5–24.9 kg/m^2^, and those with NYHA class IV, acute-phase initiation of CR may have a stronger effect on improvement of ADL.

The usefulness of acute-phase CR initiation in patients with AHF has been reported in recent years. It has been reported that acute-phase CR initiation is associated with a lower in-hospital mortality, a shorter length of hospital stays, and a lower 30-day re-admission rate due to HF of patients with AHF [5]. The REHAB-HF study reported that an early, transitional, tailored, progressive rehabilitation intervention that included multiple physical-function domains resulted in greater improvement in physical function than usual care in 349 older patients hospitalized for AHF [25]. Furthermore, the association of acute-phase CR initiation and improvement of ADL has also been reported. A study of 259 patients with AHF found that early CR was associated with improvement in return to independent functional status (unassisted walking) at the time of hospital discharge [26]. In another report, early CR is associated with the maintenance of BI in patients with AHF [27]. However, these studies consisted of a small number of cases [26], did not consider confounding factors, such as mechanical therapy and HF medications at admission [27], and excluded patients whose rehabilitation was performed on and after hospitalization day 4 (in this study, acute-phase CR initiation was defined as the initiation within 3 days after admission) [27]. To our knowledge, our study is the first large-scale evaluation of the effect of acute-phase CR initiation on ADL improvement in patients hospitalized for AHF, considering the confounding factors, and the first to examine particularly in which sub-population this effect is strong.

Our results showed that acute-phase CR initiation was associated with improvement in ADL in patients with AHF, but the effect size was smaller than anticipated. In a randomized controlled trial that examined the effects of early rehabilitation in mechanically ventilated patients, the initiation of early rehabilitation improved the return to independent ADL at hospital discharge compared to standard care (odds ratio, 2.7; 95% CI, 1.2–6.1) [23]. Additionally, in a previous study, in which mechanically ventilated patients were given 6 weeks of exercise training starting early in their hospitalization, the intervention group showed improvement in BI at 6 weeks compared to the control group (effect size, 2.02; 95% CI, 1.12–2.81) [28]. The following reasons may explain why the expected effect size was not observed in this study in contrast to the previous studies. In the previous studies, only the intervention group received rehabilitation intervention from a physical or occupational therapist, while the control group did not [23,28]. In contrast, patients in our study were classified into two groups based on whether early rehabilitation was performed or not; therefore, the non-early rehabilitation group included many patients who underwent rehabilitation after 2 or 3 days. Additionally, in our study, approximately 90% of patients were discharged home, and social factors, such as prolonged length of hospital stay and prolonged rehabilitation until ADL improved enough for discharge home, may have contributed to the effect size being smaller than expected. In fact, the non-early rehabilitation group had a significantly longer length of hospital stay compared to the early rehabilitation group. Furthermore, since this was a retrospective study, the content, frequency, intensity, and duration of early rehabilitation were not standardized among facilities or therapists.

In this study, improvement in ADL was greater in patients aged ≥80 years and in females. This may be attributed to the fact that patients aged ≥80 years had lower baseline BI than younger patients (mean baseline BI, 47 vs. 66). Similarly, females had lower baseline BI than males (mean baseline BI, 49 vs. 60), and improvement was more likely to occur with the intervention in those patients.

In recent years, the importance of tailored rehabilitation has been increasing. The REHAB-HF trial examined the effects of an early, transitional, tailored, progressive rehabilitation intervention in patients with AHF. The intervention group had significantly greater improvement in physical function at 3 months than the control group (even though 43% of the control group had undergone rehabilitation) [25]. Tailored early CR was also associated with an improved return to independent functional status (unassisted ambulation) by 30 days after admission (hazard ratio, 8.03; 95% CI, 2.15–29.98) [26]. In our study, acute-phase rehabilitation was defined only by whether or not there was an acute-phase initiation, and the association with short-term BI improvement was examined. As a result, the acute-phase initiation of rehabilitation was shown to have a beneficial effect on ADL, although its effect size was smaller than anticipated. In addition, electrical muscular stimulation therapy for leg muscles as an add-on therapy to exercise-based CR improved quadriceps strength and lower extremity function in older frail patients with AHF [29]. Hence, further research is needed into more effective modalities of intervention for patients with AHF.

Although the criteria for indication and initiation of CR in patients with AHF have not been fully investigated, recently published practical recommendations indicate that patients who do not require life support equipment or high-dose catecholamine administration because of low output syndrome and do not have untreated life-threatening cardiac arrhythmias or hemodynamic instability are currently recognized as indications for CR [30,31,32]. However, a recent study reported that patients receiving ECMO can safely undergo active physical therapy, including ambulation, when an experienced multidisciplinary team is utilized [33], and the indications for CR may expand in the future. Therefore, further data-based validation will be needed to determine the indications, contraindications, and initiation criteria for CR in patients with AHF.

There are several possible mechanisms that could explain the results obtained in this study. CR for patients with HF has been reported to improve exercise capacity [34,35], muscle fatigue [36], autonomic function [37], vascular endothelial function [38], depressive symptoms [39], and lead to left ventricular reverse remodeling [40,41]. In contrast, the adverse effects of immobility on skeletal muscles have been recognized, and the atrophy of skeletal muscles begins within 72 h [42]. Furthermore, even healthy, and well-nourished individuals can show a loss in muscle mass and strength within 10 days of bed rest [23,43]. Therefore, it is preferable to initiate rehabilitation as early as possible to obtain the most benefit from rehabilitation.

Our study has valuable clinical implications. Our study may confirm the potential association of acute-phase CR initiation with improvement of ADL in patients with AHF. Impairment of physical function and ADL in patients with AHF has recently received attention as one of the important clinical outcomes. The REHAB-HF study reported that severe functional impairments in older patients with AHF may be ameliorated by multidomain rehabilitation intervention [25,44]. These previous studies and our study indicate that CR has an important role not only in improving patients with chronic HF but also in those with AHF.

Our study had some important limitations. First, the validity of the diagnoses and procedures recorded in the database we used was reportedly high [45]. However, recorded diagnoses in databases akin to ours are generally less well-validated due to the nature of retrospective studies and administrative data. Therefore, we used propensity score matching and IPTW to adjust for confounding factors as much as possible. Nonetheless, we acknowledge that the lack of important information regarding several items, including depression, dementia, and pre-hospital ADL, which could have affected the results and could be unmeasured confounding factors, was a study limitation. Second, whereas intensive CR, a comprehensive and structured program, has been recognized as a cost-effective intervention that ensures favorable outcomes [46,47], we examined the unidentified benefits of rehabilitation from the acute phase. However, the present study did not provide detailed data on CR, including the frequency, exercise intensity, time or duration, and type of exercise. In addition, in this study, acute-phase rehabilitation was defined as when interventions were performed within 2 or 3 days of hospitalization according to previous studies [5,13,15,16], but there has not been sufficient consensus. Third, this study could not identify the cause of HF because of the nature of the nation-wide database. The causes of HF are not expected to have a significant impact on the improvement of BI, but further research on this point should be addressed in the future.

In conclusion, although our analyses of a nationwide inpatient database revealed a significant association between acute-phase CR initiation and short-term improvement in ADL in patients hospitalized for AHF, the effect size was modest. However, we discovered a sub-population whose acute-phase CR initiation may have a stronger effect on short-term improvement in ADL. Further research is needed to determine whether the effect of acute-phase CR initiation on ADL improvement is clinically meaningful.

## Figures and Tables

**Table 1:** Characteristics of patients before and after propensity score matching between patients with and without early rehabilitation (within 2 days after hospital admission).

	Before Propensity Score Matching Rehabilitation within 2 Days			After Propensity Score Matching Rehabilitation within 2 Days		
	No	Yes	SMD	No	Yes	SMD
	(*n* = 261,398)	(*n* = 45,428)		(*n* = 45,427)	(*n* = 45,427)	
Age, years	76 (±12)	79 (±11)	0.250	79 (±11)	79 (±11)	0.004
Male sex	146,421 (56.0)	23,077 (50.8)	−0.105	23,113 (50.9)	23,077 (50.8)	−0.002
Body mass index (kg/m^2^)						
<18.5	39,836 (15.2)	8187 (18.0)	0.075	8243 (18.1)	8186 (18.0)	−0.003
18.5–24.9	153,886 (58.9)	26,814 (59.0)	0.003	26,723 (58.8)	26,814 (59.0)	0.004
25.0–29.9	51,659 (19.8)	7955 (17.5)	−0.058	7912 (17.4)	7955 (17.5)	0.002
≥30.0	16,017 (6.1)	2472 (5.4)	−0.029	2549 (5.6)	2472 (5.4)	−0.007
Comorbidity						
Atrial fibrillation	104,341 (39.9)	19,319 (42.5)	0.053	19,274 (42.4)	19,318 (42.5)	0.002
Hypertension	177,608 (67.9)	31,221 (68.7)	0.017	31,342 (69.0)	31,220 (68.7)	−0.006
Diabetes mellitus	84,447 (32.3)	14,196 (31.2)	−0.023	14,246 (31.4)	14,196 (31.3)	−0.002
Chronic renal failure	38,621 (14.8)	6565 (14.5)	−0.009	6462 (14.2)	6565 (14.5)	0.006
Chronic liver disease	10,892 (4.2)	1662 (3.7)	−0.026	1681 (3.7)	1662 (3.7)	−0.002
Chronic respiratory disease	29,509 (11.3)	5716 (12.6)	0.040	5714 (12.6)	5715 (12.6)	<0.001
Anemia	40,013 (15.3)	7558 (16.6)	0.036	7478 (16.5)	7557 (16.6)	0.005
Cancer	15,886 (6.1)	2499 (5.5)	−0.025	2453 (5.4)	2499 (5.5)	0.004
Myocardial infarction	6605 (2.5)	1179 (2.6)	0.004	1186 (2.6)	1179 (2.6)	−0.001
Dilated cardiomyopathy	22,399 (8.6)	3189 (7.0)	−0.058	3164 (7.0)	3189 (7.0)	0.002
Smoking	89,868 (34.4)	14,677 (32.3)	−0.044	14,824 (32.6)	14,677 (32.3)	−0.007
Prior hospital admission	70,051 (26.8)	13,259 (29.2)	0.053	13,355 (29.4)	13,258 (29.2)	−0.005
New York Heart Association						
Class II	82,070 (31.4)	14,355 (31.6)	0.004	14,271 (31.4)	14,354 (31.6)	0.004
Class III	101,906 (39.0)	17,542 (38.6)	−0.008	17,601 (38.7)	17,542 (38.6)	−0.003
Class IV	77,422 (29.6)	13,531 (29.8)	0.004	13,555 (29.8)	13,531 (29.8)	−0.001
Barthel index at admission	62 (±39)	54 (±39)	−0.188	54 (±39)	54 (±39)	0.001
Japan Coma Scale						
0	236,146 (90.3)	39,566 (87.1)	−0.103	39,580 (87.1)	39,565 (87.1)	−0.001
1 digit	25,252 (9.7)	5862 (12.9)	0.103	5847 (12.9)	5862 (12.9)	0.001
Weekend admission	42,777 (16.4)	7459 (16.4)	0.001	7383 (16.3)	7459 (16.4)	0.005
Medication within 2 days after admission						
Beta blocker	86,132 (33.0)	17,956 (39.5)	0.137	17,904 (39.4)	17,955 (39.5)	0.002
Renin-angiotensin system inhibitor	95,746 (36.6)	18,765 (41.3)	0.096	18,885 (41.6)	18,764 (41.3)	−0.005
Mineralocorticoid receptor antagonist	82,847 (31.7)	16,356 (36.0)	0.091	16,402 (36.1)	16,355 (36.0)	−0.002
Tolvaptan	26,576 (10.2)	6818 (15.0)	0.146	6870 (15.1)	6817 (15.0)	−0.003
Intravenous inotropic agent	42,124 (16.1)	6093 (13.4)	−0.076	6119 (13.5)	6093 (13.4)	−0.002
Intravenous nitrate	49,210 (18.8)	9374 (20.6)	0.045	9423 (20.7)	9374 (20.6)	−0.003
Intravenous furosemide	168,506 (64.5)	30,182 (66.4)	0.042	30,034 (66.1)	30,181 (66.4)	0.007
Intravenous carperitide	104,223 (39.9)	18,000 (39.6)	−0.005	18,051 (39.7)	18,000 (39.6)	−0.002
Procedures within 2 days after admission						
Respiratory support	24,876 (9.5)	5383 (11.8)	0.076	5536 (12.2)	5383 (11.8)	−0.010
Hemodialysis	4697 (1.8)	464 (1.0)	−0.066	456 (1.0)	464 (1.0)	0.002
Intensive care unit stay within 2 days after admission	22,749 (8.7)	4652 (10.2)	0.053	4658 (10.3)	4652 (10.2)	<−0.001
Length of hospital stay, days *	17 (11–26)	16 (11–25)		17 (11–26)	16 (11–25)	

Data are expressed as means (±standard deviations) or numbers (percentages). SMD: Standardized mean difference. * is presented as medians (interquartile ranges), and the *p*-value was <0.001 before and after propensity score matching. The 13 variables included in the propensity score matching (10 sub-items of Barthel index, educational institute, hospital volume, and year of admission) are listed in Supplemental Table S1.

**Table 2:** Risk ratios of acute-phase initiation of cardiac rehabilitation for improving the Barthel index in the propensity score matched cohort or inverse probability of treatment weighted cohort by sensitivity analysis.

How to Account for Differences in Baseline Clinical Characteristics.	Initiation of CR			
PSM or IPTW	Within 2 Days or within 3 Days	Risk Ratio	95% CI	*p*-Value
PSM	3 days	1.028	1.016–1.040	<0.001
IPTW	2 days	1.016	1.003–1.029	0.011
IPTW	3 days	1.022	1.010–1.033	<0.001

CI: confidence interval; CR: cardiac rehabilitation; IPTW: inverse probability of treatment weighting; PSM: propensity score matching.

## Data Availability

The datasets analyzed during the current study are not publicly available due to contracts with the hospitals providing data to the database.

## Supplementary Materials

The following supporting information can be downloaded at: https://www.mdpi.com/article/10.3390/jcdd9040097/s1, Table S1: Characteristics of patients before and after propensity score matching between patients with and without early rehabilitation (within 2 days after hospital admission), Table S2: Characteristics of patients before and after propensity score matching between patients with and without early rehabilitation (within 3 days after hospital admission), Table S3: Characteristics of patients before and after inverse probability of treatment weighting between patients with and without early rehabilitation (within 2 days after hospital admission), Table S4: Characteristics of patients before and after inverse probability of treatment weighting between patients with and without early rehabilitation (within 3 days after hospital admission).
