# Supplementary material for: Acute-Phase Initiation of Cardiac Rehabilitation for Short-Term Improvement in Activities of Daily Living in Patients Hospitalized for Acute Heart Failure
Source: J Cardiovasc Dev Dis. 2022 Mar 25;9(4):97. doi: 10.3390/jcdd9040097 (PMC9025467; doi:10.3390/jcdd9040097)
Supplement: Supplementary file 1 [file jcdd-09-00097-s001.zip › jcdd-1608450-supplementary.pdf]

Supplemental Table S1: Characteristics of patients with and without early rehabilitation (within 2 days after hospital admission) before and after propensity score matching

	Before propensity score matching Rehabilitation within 2 days				After propensity score matching Rehabilitation within 2 days		
	No	Yes	P-value	SMD	No	Yes	SMD
	(n=261,398)	(n=45,428)			(n=45,427)	(n=45,427)	
Age, years	76 (±12)	79 (±11)	<0.001	0.250	79 (±11)	79 (±11)	0.004
Male sex	146,421 (56.0)	23,077 (50.8)	<0.001	-0.105	23,113 (50.9)	23,077 (50.8)	-0.002
Body mass index (kg/m^2)			<0.001				
<18.5	39,836 (15.2)	8,187 (18.0)		0.075	8,243 (18.1)	8,186 (18.0)	-0.003
18.5–24.9	153,886 (58.9)	26,814 (59.0)		0.003	26,723 (58.8)	26,814 (59.0)	0.004
25.0–29.9	51659 (19.8)	7,955 (17.5)		-0.058	7,912 (17.4)	7,955 (17.5)	0.002
≥30.0	16,017 (6.1)	2,472 (5.4)		-0.029	2,549 (5.6)	2,472 (5.4)	-0.007
Comorbidity							
Atrial fibrillation	104,341 (39.9)	19,319 (42.5)	<0.001	0.053	19,274 (42.4)	19,318 (42.5)	0.002
Hypertension	177,608 (67.9)	31,221 (68.7)	<0.001	0.017	31,342 (69.0)	31,220 (68.7)	-0.006
Diabetes mellitus	84,447 (32.3)	14,196 (31.2)	<0.001	-0.023	14,246 (31.4)	14,196 (31.3)	-0.002
Chronic renal failure	38,621 (14.8)	6,565 (14.5)	0.073	-0.009	6,462 (14.2)	6,565 (14.5)	0.006
Chronic liver disease	10,892 (4.2)	1,662 (3.7)	<0.001	-0.026	1,681 (3.7)	1,662 (3.7)	-0.002
Chronic respiratory disease	29,509 (11.3)	5,716 (12.6)	<0.001	0.040	5,714 (12.6)	5,715 (12.6)	<0.001
Anemia	40,013 (15.3)	7,558 (16.6)	<0.001	0.036	7,478 (16.5)	7,557 (16.6)	0.005
Cancer	15,886 (6.1)	2,499 (5.5)	<0.001	-0.025	2,453 (5.4)	2,499 (5.5)	0.004
Myocardial infarction	6,605 (2.5)	1,179 (2.6)	0.390	0.004	1,186 (2.6)	1,179 (2.6)	-0.001
Dilated cardiomyopathy	22,399 (8.6)	3,189 (7.0)	<0.001	-0.058	3,164 (7.0)	3,189 (7.0)	0.002
Smoking	89,868 (34.4)	14,677 (32.3)	<0.001	-0.044	14,824 (32.6)	14,677 (32.3)	-0.007

Prior hospital admission	70,051 (26.8)	13,259 (29.2)	<0.001	0.053	13,355 (29.4)	13,258 (29.2)	-0.005
New York Heart Association			0.330				
Class II	82,070 (31.4)	14,355 (31.6)		0.004	14,271 (31.4)	14,354 (31.6)	0.004
Class III	101,906 (39.0)	17,542 (38.6)		-0.008	17,601 (38.7)	17,542 (38.6)	-0.003
Class IV	77,422 (29.6)	13,531 (29.8)		0.004	13,555 (29.8)	13,531 (29.8)	-0.001
Total Barthel Index score at admission	62 (±39)	54 (±39)	<0.001	-0.188	54 (±39)	54 (±39)	0.001
Barthel index: Bowels			<0.001				
0	63,153 (24.2)	13,350 (29.4)		0.118	13,502 (29.7)	13,350 (29.4)	-0.007
5	34,127 (13.1)	6,972 (15.3)		0.066	6,754 (14.9)	6,971 (15.3)	0.013
10	164,118 (62.8)	25,106 (55.3)		-0.153	25,171 (55.4)	25,106 (55.3)	-0.003
Barthel index: Bladder			<0.001				
0	66,981 (25.6)	14,091 (31.0)		0.120	14,196 (31.3)	14,091 (31.0)	-0.005
5	33,529 (12.8)	6,834 (15.0)		0.064	6,648 (14.6)	6,833 (15.0)	0.011
10	160,888 (61.5)	24,503 (53.9)		-0.155	24,583 (54.1)	24,503 (53.9)	-0.004
Barthel index: Grooming			<0.001				
0	110,882 (42.4)	23,286 (51.3)		0.178	23,256 (51.2)	23,285 (51.3)	0.001
5	150,516 (57.6)	22,142 (48.7)		-0.178	22,171 (48.8)	22,142 (48.7)	-0.001
Barthel index: Toilet Use			<0.001				
0	71,286 (27.3)	15,331 (33.7)		0.141	15,384 (33.9)	15,331 (33.7)	-0.002
5	56,527 (21.6)	11,076 (24.4)		0.066	10,922 (24.0)	11,075 (24.4)	0.008
10	133,585 (51.1)	19,021 (41.9)		-0.186	19,121 (42.1)	19,021 (41.9)	-0.004
Barthel index: Feeding			<0.001				
0	47,291 (18.1)	9,613 (21.2)		0.077	9,683 (21.3)	9,613 (21.2)	-0.004

5	47,803 (18.3)	10,093 (22.2)		0.098	10,024 (22.1)	10,092 (22.2)	0.004
10	166,304 (63.6)	25,722 (56.6)		-0.143	25,720 (56.6)	25,722 (56.6)	<0.001
Barthel index: Transfer			<0.001				
0	58,659 (22.4)	12,483 (27.5)		0.117	12,504 (27.5)	12,483 (27.5)	-0.001
5	18,964 (7.3)	3,805 (8.4)		0.042	3,880 (8.5)	3,805 (8.4)	-0.006
10	56,794 (21.7)	11,264 (24.8)		0.073	11,109 (24.5)	11,263 (24.8)	0.008
15	126,981 (48.6)	17,876 (39.4)		-0.187	17,934 (39.5)	17,876 (39.4)	-0.003
Barthel index: Mobility			<0.001				
0	83,895 (32.1)	18,091 (39.8)		0.162	18,106 (39.9)	18,090 (39.8)	-0.001
5	20,694 (7.9)	3,567 (7.9)		-0.002	3,594 (7.9)	3,567 (7.9)	-0.002
10	32,445 (12.4)	6,486 (14.3)		0.055	6,408 (14.1)	6,486 (14.3)	0.005
15	124,364 (47.6)	17,284 (38.0)		-0.193	17,319 (38.1)	17,284 (38.0)	-0.002
Barthel index: Dressing			<0.001				
0	68,851 (26.3)	14,712 (32.4)		0.133	14,853 (32.7)	14,712 (32.4)	-0.007
5	64,340 (24.6)	12,532 (27.6)		0.068	12,338 (27.2)	12,531 (27.6)	0.010
10	128, 207 (49.0)	18,184 (40.0)		-0.182	18,236 (40.1)	18,184 (40.0)	-0.002
Barthel index: Stairs			<0.001				
0	104,059 (39.8)	22,099 (48.6)		0.179	22,216 (48.9)	22,098 (48.6)	-0.005
5	41,527 (15.9)	7,544 (16.6)		0.020	7,387 (16.3)	7,544 (16.6)	0.009
10	115,812 (44.3)	15,785 (34.7)		-0.196	15,824 (34.8)	15,785 (34.7)	-0.002
Barthel index: Bathing			<0.001				
0	138,671 (53.0)	28,406 (62.5)		0.193	28,405 (62.5)	28,405 (62.5)	<0.001
5	122,727 (47.0)	17,022 (37.5)		-0.193	17,022 (37.5)	17,022 (37.5)	<0.001

Japan Coma Scale			<0.001				
0	236,146 (90.3)	39,566 (87.1)		-0.103	39,580 (87.1)	39,565 (87.1)	-0.001
1 digit	25,252 (9.7)	5,862 (12.9)		0.103	5,847 (12.9)	5,862 (12.9)	0.001
Weekend Admission	42,777 (16.4)	7,459 (16.4)	0.770	0.001	7,383 (16.3)	7,459 (16.4)	0.005
Medication within 2 days after admission							
Beta blocker	86,132 (33.0)	17,956 (39.5)	<0.001	0.137	17,904 (39.4)	17,955 (39.5)	0.002
Renin-angiotensin system inhibitor	95,746 (36.6)	18,765 (41.3)	<0.001	0.096	18,885 (41.6)	18,764 (41.3)	-0.005
Mineralocorticoid receptor antagonist	82,847 (31.7)	16,356 (36.0)	<0.001	0.091	16,402 (36.1)	16,355 (36.0)	-0.002
Tolvaptan	26,576 (10.2)	6,818 (15.0)	<0.001	0.146	6,870 (15.1)	6,817 (15.0)	-0.003
Intravenous inotropic agent	42,124 (16.1)	6,093 (13.4)	<0.001	-0.076	6119 (13.5)	6,093 (13.4)	-0.002
Intravenous nitrate	49,210 (18.8)	9,374 (20.6)	<0.001	0.045	9,423 (20.7)	9,374 (20.6)	-0.003
Intravenous furosemide	168,506 (64.5)	30,182 (66.4)	<0.001	0.042	30,034 (66.1)	30,181 (66.4)	0.007
Intravenous carperitide	104,223 (39.9)	18,000 (39.6)	0.320	-0.005	18,051 (39.7)	18,000 (39.6)	-0.002
Procedures within 2 days after admission							
Respiratory support	24,876 (9.5)	5,383 (11.8)	<0.001	0.076	5,536 (12.2)	5,383 (11.8)	-0.010
Hemodialysis	4,697 (1.8)	464 (1.0)	<0.001	-0.066	456 (1.0)	464 (1.0)	0.002
Intensive care unit stay within 2 days after admission	22,749 (8.7)	4,652 (10.2)	<0.001	0.053	4,658 (10.3)	4,652 (10.2)	<-0.001
Educational institute	206,886 (79.1)	36,189 (79.7)	0.012	0.013	35,989 (79.2)	36,189 (79.7)	0.011
hospital volume			<0.001				
Low	89,376 (34.2)	12,753 (28.1)		-0.132	12,729 (28.0)	12,753 (28.1)	0.001
Medium	87,056 (33.3)	14,530 (32.0)		-0.028	14,566 (32.1)	14,530 (32.0)	-0.002
High	84,966 (32.5)	18,145 (39.9)		0.155	18,132 (39.9)	18,144 (39.9)	0.001
Year of admission (year)			<0.001				

2010	16,483 (6.3)	1,281 (2.8)	-0.168	1,309 (2.9)	1,281 (2.8)	-0.004
2011	37,489 (14.3)	3,142 (6.9)	-0.243	3,201 (7.0)	3,142 (6.9)	-0.005
2012	40,485 (15.5)	4,466 (9.8)	-0.171	4,471 (9.8)	4,466 (9.8)	<-0.001
2013	40,102 (15.3)	5,220 (11.5)	-0.113	5,125 (11.3)	5,220 (11.5)	0.007
2014	41,612 (15.9)	7,701 (17.0)	0.028	7,674 (16.9)	7,701 (17.0)	0.002
2015	43,993 (16.8)	10,670 (23.5)	0.167	10,647 (23.4)	10,670 (23.5)	0.001
2016	24,177 (9.2)	7,041 (15.5)	0.191	7,092 (15.6)	7,040 (15.5)	-0.003
2017	14,070 (5.4)	4,863 (10.7)	0.197	4,862 (10.7)	4,863 (10.7)	<0.001
2018	2,987 (1.1)	1,044 (2.3)	0.089	1,046 (2.3)	1,044 (2.3)	<-0.001

Data are expressed as means (± standard deviations) or numbers (percentages).
SMD, standardized mean difference

Supplemental Table S2: Characteristics of patients with and without early rehabilitation (within 3 days after hospital admission) before and after propensity score matching

	Before propensity score matching Rehabilitation within 3 days				After propensity score matching Rehabilitation within 3 days		
	No	Yes	P-value	SMD	No	Yes	SMD
	(n=242,181)	(n=60,359)			(n=60,358)	(n=60,358)	
Age, years	76 (±12)	79 (±11)	<0.001	0.258	79 (±11)	79 (±11)	0.002
Male sex	136,292 (56.3)	30,670 (50.8)	<0.001	-0.110	30,692 (50.8)	30,670 (50.8)	-0.001
Body mass index (kg/m^2)			<0.001				
<18.5	36,506 (15.1)	10,881 (18.0)		0.080	10,840 (18.0)	10,880 (18.0)	0.002
18.5–24.9	142,492 (58.8)	35,632 (59.0)		0.004	35,647 (59.1)	35,632 (59.0)	-0.001
25.0–29.9	48,203 (19.9)	10,577 (17.5)		-0.061	10,514 (17.4)	10,577 (17.5)	0.003
≥30.0	14,980 (6.2)	3,269 (5.4)		-0.033	3,357 (5.6)	3,269 (5.4)	-0.006
Comorbidity							
Atrial fibrillation	96,668 (39.9)	25,560 (42.3)	<0.001	0.049	25,357 (42.0)	25,560 (42.3)	0.007
Hypertension	164,439 (67.9)	41,732 (69.1)	<0.001	0.027	41,919 (69.5)	41,731 (69.1)	-0.007
Diabetes mellitus	78,486 (32.4)	18,934 (31.4)	<0.001	-0.022	19,152 (31.7)	18,934 (31.4)	-0.008
Chronic renal failure	35,714 (14.7)	8,822 (14.6)	0.420	-0.004	8,784 (14.6)	8,822 (14.6)	0.002
Chronic liver disease	10,202 (4.2)	2,182 (3.6)	<0.001	-0.031	2,242 (3.7)	2,182 (3.6)	-0.005
Chronic respiratory disease	27,287 (11.3)	7,493 (12.4)	<0.001	0.036	7,491 (12.4)	7,493 (12.4)	<0.001
Anemia	36,979 (15.3)	10,146 (16.8)	<0.001	0.042	10,205 (16.9)	10,146 (16.8)	-0.003
Cancer	14,809 (6.1)	3,377 (5.6)	<0.001	-0.022	3,457 (5.7)	3,377 (5.6)	-0.006
Myocardial infarction	6,121 (2.5)	1,551 (2.6)	0.560	0.003	1,596 (2.6)	1,551 (2.6)	-0.005
Dilated cardiomyopathy	20,975 (8.7)	4,293 (7.1)	<0.001	-0.057	4,208 (7.0)	4,293 (7.1)	0.006
Smoking	83,533 (34.5)	19,462 (32.2)	<0.001	-0.048	19,530 (32.4)	19,462 (32.2)	-0.002

Prior hospital admission	64,740 (26.7)	17,429 (28.9)	<0.001	0.048	17,569 (29.1)	17,429 (28.9)	-0.005
New York Heart Association			0.002				
Class II	75,899 (31.3)	18,669 (30.9)		-0.009	18,667 (30.9)	18,669 (30.9)	<0.001
Class III	94,710 (39.1)	23,407 (38.8)		-0.007	23,280 (38.6)	23,406 (38.8)	0.004
Class IV	71,572 (29.6)	18,283 (30.3)		0.016	18,411 (30.5)	18,283 (30.3)	-0.005
Total Barthel Index score at admission	62 (±39)	54 (±39)	<0.001	-0.211	54 (±39)	54 (±39)	0.001
Barthel index: Bowels			<0.001				
0	57,520 (23.8)	18,044 (29.9)		0.139	18,163 (30.1)	18,043 (29.9)	-0.004
5	31,456 (13.0)	9,178 (15.2)		0.064	9,057 (15.0)	9,178 (15.2)	0.006
10	153,205 (63.3)	33,137 (54.9)		-0.171	33,138 (54.9)	33,137 (54.9)	<-0.001
Barthel index: Bladder			<0.001				
0	61,032 (25.2)	19,047 (31.6)		0.141	19,156 (31.7)	19,046 (31.6)	-0.004
5	30,916 (12.8)	8,985 (14.9)		0.061	8,888 (14.7)	8,985 (14.9)	0.005
10	150,233 (62.0)	32,327 (53.6)		-0.172	32,314 (53.5)	32,327 (53.6)	<0.001
Barthel index: Grooming			<0.001				
0	101,514 (41.9)	31,099 (51.5)		0.193	31,102 (51.5)	31,098 (51.5)	<-0.001
5	140,667 (58.1)	29,260 (48.5)		-0.193	29,256 (48.5)	29,260 (48.5)	<0.001
Barthel index: Toilet Use			<0.001				
0	64,878 (26.8)	20,712 (34.3)		0.164	20,769 (34.4)	20,711 (34.3)	-0.002
5	52,255 (21.6)	14,565 (24.1)		0.061	14,446 (23.9)	14,565 (24.1)	0.005
10	125,048 (51.6)	25,082 (41.6)		-0.203	25,143 (41.7)	25,082 (41.6)	-0.002
Barthel index: Feeding			<0.001				
0	43,024 (17.8)	13,158 (21.8)		0.101	13,272 (22.0)	13,158 (21.8)	-0.005

5	43,919 (18.1)	13,348 (22.1)		0.099	13,238 (21.9)	13,348 (22.1)	0.004
10	155,238 (64.1)	33,853 (56.1)		-0.164	33,848 (56.1)	33,852 (56.1)	<0.001
Barthel index: Transfer			<0.001				
0	53,286 (22.0)	16,977 (28.1)		0.142	17,029 (28.2)	16,977 (28.1)	-0.002
5	17,417 (7.2)	5,087 (8.4)		0.046	5,139 (8.5)	5,087 (8.4)	-0.003
10	52,551 (21.7)	14,758 (24.5)		0.065	14,587 (24.2)	14,757 (24.4)	0.007
15	118,927 (49.1)	23,537 (39.0)		-0.205	23,603 (39.1)	23,537 (39.0)	-0.002
Barthel index: Mobility			<0.001				
0	76,413 (31.6)	24,370 (40.4)		0.185	24,364 (40.4)	24,369 (40.4)	<0.001
5	19,300 (8.0)	4,696 (7.8)		-0.007	4,684 (7.8)	4,696 (7.8)	0.001
10	29,936 (12.4)	8,524 (14.1)		0.052	8,528 (14.1)	8,524 (14.1)	<-0.001
15	116,532 (48.1)	22,769 (37.7)		-0.211	22,782 (37.7)	22,769 (37.7)	<-0.001
Barthel index: Dressing			<0.001				
0	62,675 (25.9)	19,873 (32.9)		0.155	19,942 (33.0)	19,872 (32.9)	-0.002
5	59,475 (24.6)	16,541 (27.4)		0.065	16,412 (27.2)	16,541 (27.4)	0.005
10	120,031 (49.6)	23,945 (39.7)		-0.200	24,004 (39.8)	23,945 (39.7)	-0.002
Barthel index: Stairs			<0.001				
0	95,147 (39.3)	29,531 (48.9)		0.195	29,509 (48.9)	29,530 (48.9)	0.001
5	38,526 (15.9)	9,953 (16.5)		0.016	9,883 (16.4)	9,953 (16.5)	0.003
10	108,508 (44.8)	20,875 (34.6)		-0.210	20,966 (34.7)	20,875 (34.6)	-0.003
Barthel index: Bathing			<0.001				
0	127,332 (52.6)	37,809 (62.6)		0.205	37,774 (62.6)	37,808 (62.6)	0.001
5	114,849 (47.4)	22,550 (37.4)		-0.205	22,584 (37.4)	22,550 (37.4)	-0.001

Japan Coma Scale			<0.001				
0	219,260 (90.5)	52,645 (87.2)		-0.106	52,575 (87.1)	52,644 (87.2)	0.003
1 digit	22,921 (9.5)	7,714 (12.8)		0.106	7,783 (12.9)	7,714 (12.8)	-0.003
Weekend Admission	38,146 (15.8)	11,317 (18.7)	<0.001	0.079	11,346 (18.8)	11,316 (18.7)	-0.001
Medication within 2 days after admission							
Beta blocker	79,254 (32.7)	23,513 (39.0)	<0.001	0.130	23,509 (38.9)	23,513 (39.0)	<0.001
Renin-angiotensin system inhibitor	88,472 (36.5)	24,785 (41.1)	<0.001	0.093	24,939 (41.3)	24,784 (41.1)	-0.005
Mineralocorticoid receptor antagonist	76,727 (31.7)	21,393 (35.4)	<0.001	0.080	21,427 (35.5)	21,393 (35.4)	-0.001
Tolvaptan	24,247 (10.0)	8,764 (14.5)	<0.001	0.138	8,812 (14.6)	8,763 (14.5)	-0.002
Intravenous inotropic agent	39,340 (16.2)	8,273 (13.7)	<0.001	-0.071	8,190 (13.6)	8,273 (13.7)	0.004
Intravenous nitrate	44,447 (18.4)	12,858 (21.3)	<0.001	0.074	12,902 (21.4)	12,858 (21.3)	-0.002
Intravenous furosemide	156,071 (64.4)	40,709 (67.4)	<0.001	0.063	40,489 (67.1)	40,709 (67.4)	0.008
Intravenous carperitide	96,686 (39.9)	24,568 (40.7)	<0.001	0.016	24,646 (40.8)	24,568 (40.7)	-0.003
Procedures within 2 days after admission							
Respiratory support	22,555 (9.3)	7,358 (12.2)	<0.001	0.093	7,454 (12.3)	7,358 (12.2)	-0.005
Hemodialysis	4,264 (1.8)	653 (1.1)	<0.001	-0.057	658 (1.1)	653 (1.1)	-0.001
Intensive care unit stay within 2 days after admission	20,634 (8.5)	6,486 (10.7)	<0.001	0.075	6,528 (10.8)	6,486 (10.7)	-0.002
Educational institute	191,353 (79.0)	48,361 (80.1)	<0.001	0.028	48,129 (79.7)	48,360 (80.1)	0.010
hospital volume			<0.001				
Low	83,436 (34.5)	16,992 (28.2)		-0.136	17,033 (28.2)	16,992 (28.2)	-0.002
Medium	81,357 (33.6)	19,830 (32.9)		-0.016	19,924 (33.0)	19,830 (32.9)	-0.003
High	77,388 (32.0)	23,537 (39.0)		0.148	2,3401 (38.8)	23,536 (39.0)	0.005
Year of admission (year)			<0.001				

2010	15,786 (6.5)	1,755 (2.9)	-0.171	1,751 (2.9)	1,755 (2.9)	<0.001
2011	35,665 (14.7)	4,363 (7.2)	-0.242	4,445 (7.4)	4,363 (7.2)	-0.005
2012	38,174 (15.8)	6,135 (10.2)	-0.167	6,079 (10.1)	6,135 (10.2)	0.003
2013	37,469 (15.5)	7,213 (12.0)	-0.103	7,186 (11.9)	7,213 (12.0)	0.001
2014	38,499 (15.9)	10,146 (16.8)	0.025	10,043 (16.6)	10,146 (16.8)	0.005
2015	39,937 (16.5)	13,968 (23.1)	0.167	13,909 (23.0)	13,968 (23.1)	0.002
2016	21,610 (8.9)	9,178 (15.2)	0.194	9,364 (15.5)	9,178 (15.2)	-0.009
2017	12,430 (5.1)	6,241 (10.3)	0.196	6,215 (10.3)	6,240 (10.3)	0.001
2018	2,611 (1.1)	1,360 (2.3)	0.092	1,366 (2.3)	1,360 (2.3)	-0.001

Data are expressed as means (± standard deviations) or numbers (percentages).
SMD, standardized mean difference

Supplemental Table S3: Characteristics of patients with and without early rehabilitation before and after inverse probability of treatment weighting (within 2 days after hospital admission)

	Before IPTWs weighted Rehabilitation within 2 days			After IPTWs weighted Rehabilitation within 2 days		
	No	Yes	SMD	No	Yes	SMD
	(n=261,398)	(n=45,428)		(n=261,398)	(n=45,428)	
Age, years	76 (±12)	79 (±11)	0.250	77 (±12)	77 (±12)	-0.006
Male sex	56.0	50.8	-0.105	55.2	55.2	<-0.001
Body mass index (kg/m^2)						
<18.5	15.2	18.0	0.075	15.7	15.8	0.003
18.5–24.9	58.9	59.0	0.003	58.9	58.8	-0.001
25.0–29.9	19.8	17.5	-0.058	19.4	19.3	-0.004
≥30.0	6.1	5.4	-0.029	6.0	6.2	0.005
Comorbidity						
Atrial fibrillation	39.9	42.5	0.053	40.3	39.9	-0.008
Hypertension	67.9	68.7	0.017	68.1	68.0	-0.001
Diabetes mellitus	32.3	31.2	-0.023	32.2	32.5	0.008
Chronic renal failure	14.8	14.5	-0.009	14.7	14.6	-0.003
Chronic liver disease	4.2	3.7	-0.026	4.1	4.1	-0.001
Chronic respiratory disease	11.3	12.6	0.040	11.5	11.6	0.004
Anemia	15.3	16.6	0.036	15.5	15.6	0.002
Cancer	6.1	5.5	-0.025	6.0	5.8	-0.007
Myocardial infarction	2.5	2.6	0.004	2.5	2.7	0.007
Dilated cardiomyopathy	8.6	7.0	-0.058	8.3	8.4	0.002

Smoking	34.4	32.3	-0.044	34.1	34.3	0.005
Prior hospital admission	26.8	29.2	0.053	27.2	27.7	0.011
New York Heart Association						
Class II	31.4	31.6	0.004	31.4	31.3	-0.002
Class III	39.0	38.6	-0.008	38.9	38.6	-0.007
Class IV	29.6	29.8	0.004	29.7	30.1	0.010
Total Barthel Index score at admission	62 (±39)	54 (±39)	-0.188	61 (±39)	60 (±39)	-0.007
Barthel index: Bowels						
0	24.2	29.4	0.118	25.0	25.3	0.009
5	13.1	15.3	0.066	13.4	13.4	0.001
10	62.8	55.3	-0.153	61.7	61.2	-0.008
Barthel index: Bladder						
0	25.6	31.0	0.120	26.4	26.7	0.007
5	12.8	15.0	0.064	13.2	13.2	0.003
10	61.5	53.9	-0.155	60.4	60.0	-0.008
Barthel index: Grooming						
0	42.4	51.3	0.178	43.7	44.0	0.005
5	57.6	48.7	-0.178	56.3	56.0	-0.005
Barthel index: Toilet Use						
0	27.3	33.7	0.141	28.3	28.5	0.005
5	21.6	24.4	0.066	22.0	21.9	-0.003
10	51.1	41.9	-0.186	49.7	49.6	-0.002
Barthel index: Feeding						
0	18.1	21.2	0.077	18.6	18.9	0.008

5	18.3	22.2	0.098	18.9	18.8	-0.001
10	63.6	56.6	-0.143	62.6	62.3	-0.006
Barthel index: Transfer						
0	22.4	27.5	0.117	23.2	23.3	0.003
5	7.3	8.4	0.042	7.4	7.5	0.005
10	21.7	24.8	0.073	22.2	22.0	-0.003
15	48.6	39.4	-0.187	47.2	47.1	-0.002
Barthel index: Mobility						
0	32.1	39.8	0.162	33.3	33.5	0.005
5	7.9	7.9	-0.002	7.9	7.9	0.001
10	12.4	14.3	0.055	12.7	12.6	-0.001
15	47.6	38.0	-0.193	46.2	45.9	-0.005
Barthel index: Dressing						
0	26.3	32.4	0.133	27.3	27.5	0.005
5	24.6	27.6	0.068	25.0	25.0	-0.001
10	49.0	40.0	-0.182	47.7	47.5	-0.003
Barthel index: Stairs						
0	39.8	48.6	0.179	41.1	41.5	0.007
5	15.9	16.6	0.020	16.0	15.9	-0.002
10	44.3	34.7	-0.196	42.9	42.6	-0.006
Barthel index: Bathing						
0	53.0	62.5	0.193	54.5	54.8	0.007
5	47.0	37.5	-0.193	45.5	45.2	-0.007
Japan Coma Scale						

0	90.3	87.1	-0.103	89.8	89.7	-0.004
1 digit	9.7	12.9	0.103	10.2	10.3	0.004
Weekend Admission	16.4	16.4	0.001	16.4	16.4	0.002
Medication within 2 days after admission						
Beta blocker	33.0	39.5	0.137	33.9	34.2	0.005
Renin-angiotensin system inhibitor	36.6	41.3	0.096	37.3	37.2	-0.003
Mineralocorticoid receptor antagonist	31.7	36.0	0.091	32.3	31.7	-0.013
Tolvaptan	10.2	15.0	0.146	10.9	10.9	-0.001
Intravenous inotropic agent	16.1	13.4	-0.076	15.7	15.5	-0.007
Intravenous nitrate	18.8	20.6	0.045	19.1	18.9	-0.005
Intravenous furosemide	64.5	66.4	0.042	64.7	63.7	-0.022
Intravenous carperitide	39.9	39.6	-0.005	39.9	40.2	0.008
Procedures within 2 days after admission						
Respiratory support	9.5	11.8	0.076	9.9	10.3	0.013
Hemodialysis	1.8	1.0	-0.066	1.7	1.7	0.002
Intensive care unit stay within 2 days after admission	8.7	10.2	0.053	9.0	9.4	0.015
Educational institute	79.1	79.7	0.013	79.2	78.6	-0.016
hospital volume						
Low	34.2	28.1	-0.132	33.3	33.7	0.009
Medium	33.3	32.0	-0.028	33.1	33.2	0.003
High	32.5	39.9	0.155	33.6	33.1	-0.011
Year of admission (year)						
2010	6.3	2.8	-0.168	5.8	5.9	0.004

2011	14.3	6.9	-0.243	13.2	13.4	0.004
2012	15.5	9.8	-0.171	14.7	14.6	<-0.001
2013	15.3	11.5	-0.113	14.8	14.6	-0.004
2014	15.9	17.0	0.028	16.1	16.0	-0.001
2015	16.8	23.5	0.167	17.8	17.8	-0.001
2016	9.2	15.5	0.191	10.2	10.2	-0.001
2017	5.4	10.7	0.197	6.2	6.2	0.001
2018	1.1	2.3	0.089	1.3	1.3	0.002

Data are expressed as means (± standard deviations) or percentages.
IPTW, inverse probability of treatment weighting; SMD, standardized mean difference

Supplemental Table S4: Characteristics of patients with and without early rehabilitation before and after inverse probability of treatment weighting (within 3 days after hospital admission)

	Before IPTWs weighted Rehabilitation within 3 days			After IPTWs weighted Rehabilitation within 3 days		
	No	Yes	SMD	No	Yes	SMD
	(n=242,181)	(n=60,359)		(n=242,181)	(n=60,359)	
Age, years	76 (±12)	79 (±11)	0.258	77 (±12)	77 (±12)	-0.006
Male sex	56.3	50.8	-0.110	55.2	55.2	<0.001
Body mass index (kg/m^2)						
<18.5	15.1	18.0	0.080	15.7	15.8	0.003
18.5–24.9	58.8	59.0	0.004	58.9	58.8	-0.002
25.0–29.9	19.9	17.5	-0.061	19.4	19.3	-0.002
≥30.0	6.2	5.4	-0.033	6.0	6.1	0.004
Comorbidity						
Atrial fibrillation	39.9	42.3	0.049	40.4	40.0	-0.009
Hypertension	67.9	69.1	0.027	68.2	68.0	-0.003
Diabetes mellitus	32.4	31.4	-0.022	32.2	32.5	0.006
Chronic renal failure	14.7	14.6	-0.004	14.7	14.7	<-0.001
Chronic liver disease	4.2	3.6	-0.031	4.1	4.0	-0.002
Chronic respiratory disease	11.3	12.4	0.036	11.5	11.6	0.004
Anemia	15.3	16.8	0.042	15.6	15.6	-0.001
Cancer	6.1	5.6	-0.022	6.0	5.9	-0.006
Myocardial infarction	2.5	2.6	0.003	2.5	2.6	0.006
Dilated cardiomyopathy	8.7	7.1	-0.057	8.4	8.4	0.002

Smoking	34.5	32.2	-0.048	34.1	34.2	0.004
Prior hospital admission	26.7	28.9	0.048	27.2	27.5	0.008
New York Heart Association						
Class II	31.3	30.9	-0.009	31.2	31.2	-0.002
Class III	39.1	38.8	-0.007	39.0	38.7	-0.007
Class IV	29.6	30.3	0.016	29.7	30.2	0.010
Total Barthel Index score at admission	62 (±39)	54 (±39)	-0.211	61 (±39)	60 (±39)	-0.007
Barthel index: Bowels						
0	23.8	29.9	0.139	25.0	25.4	0.008
5	13.0	15.2	0.064	13.4	13.4	<-0.001
10	63.3	54.9	-0.171	61.6	61.2	-0.007
Barthel index: Bladder						
0	25.2	31.6	0.141	26.5	26.8	0.006
5	12.8	14.9	0.061	13.2	13.2	0.001
10	62.0	53.6	-0.172	60.3	60.0	-0.007
Barthel index: Grooming						
0	41.9	51.5	0.193	43.9	44.1	0.006
5	58.1	48.5	-0.193	56.1	55.9	-0.006
Barthel index: Toilet Use						
0	26.8	34.3	0.164	28.3	28.6	0.005
5	21.6	24.1	0.061	22.1	22.0	-0.002
10	51.6	41.6	-0.203	49.6	49.4	-0.003
Barthel index: Feeding						

0	17.8	21.8	0.101	18.6	18.9	0.008
5	18.1	22.1	0.099	18.9	18.9	-0.001
10	64.1	56.1	-0.164	62.5	62.2	-0.005
Barthel index: Transfer						
0	22.0	28.1	0.142	23.3	23.4	0.004
5	7.2	8.4	0.046	7.4	7.6	0.004
10	21.7	24.5	0.065	22.2	22.1	-0.003
15	49.1	39.0	-0.205	47.1	46.9	-0.002
Barthel index: Mobility						
0	31.6	40.4	0.185	33.4	33.6	0.006
5	8.0	7.8	-0.007	7.9	7.9	<-0.001
10	12.4	14.1	0.052	12.7	12.6	-0.002
15	48.1	37.7	-0.211	46.0	45.8	-0.005
Barthel index: Dressing						
0	25.9	32.9	0.155	27.3	27.6	0.006
5	24.6	27.4	0.065	25.1	25.0	-0.002
10	49.6	39.7	-0.200	47.6	47.4	-0.004
Barthel index: Stairs						
0	39.3	48.9	0.195	41.3	41.6	0.007
5	15.9	16.5	0.016	16.0	15.9	-0.002
10	44.8	34.6	-0.210	42.7	42.5	-0.005
Barthel index: Bathing						
0	52.6	62.6	0.205	54.6	54.9	0.006

5	47.4	37.4	-0.205	45.4	45.1	-0.006
Japan Coma Scale						
0	90.5	87.2	-0.106	89.9	89.7	-0.004
1 digit	9.5	12.8	0.106	10.1	10.3	0.004
Weekend Admission	15.8	18.7	0.079	16.4	16.4	0.002
Medication within 2 days after admission						
Beta blocker	32.7	39.0	0.130	34.0	34.1	0.003
Renin-angiotensin system inhibitor	36.5	41.1	0.093	37.4	37.3	-0.002
Mineralocorticoid receptor antagonist	31.7	35.4	0.080	32.4	31.9	-0.010
Tolvaptan	10.0	14.5	0.138	10.9	10.9	-0.002
Intravenous inotropic agent	16.2	13.7	-0.071	15.7	15.6	-0.004
Intravenous nitrate	18.4	21.3	0.074	18.9	18.8	-0.002
Intravenous furosemide	64.4	67.4	0.063	65.0	64.1	-0.018
Intravenous carperitide	39.9	40.7	0.016	40.1	40.5	0.007
Procedures within 2 days after admission						
Respiratory support	9.3	12.2	0.093	9.9	10.3	0.013
Hemodialysis	1.8	1.1	-0.057	1.6	1.7	0.004
Intensive care unit stay within 2 days after admission	8.5	10.7	0.075	9.0	9.3	0.011
Educational institute	79.0	80.1	0.028	79.2	78.7	-0.013
hospital volume						
Low	34.5	28.2	-0.136	33.2	33.6	0.009
Medium	33.6	32.9	-0.016	33.4	33.5	0.001
High	32.0	39.0	0.148	33.3	32.9	-0.010

Year of admission (year)						
2010	6.5	2.9	-0.171	5.8	5.9	0.003
2011	14.7	7.2	-0.242	13.2	13.3	0.002
2012	15.8	10.2	-0.167	14.6	14.6	-0.001
2013	15.5	12.0	-0.103	14.8	14.7	-0.001
2014	15.9	16.8	0.025	16.1	16.0	-0.002
2015	16.5	23.1	0.167	17.8	17.8	<0.001
2016	8.9	15.2	0.194	10.2	10.2	-0.001
2017	5.1	10.3	0.196	6.2	6.2	<0.001
2018	1.1	2.3	0.092	1.3	1.3	0.001

Data are expressed as means (± standard deviations) or percentages.
IPTW, inverse probability of treatment weighting; SMD, standardized mean difference
